# Audit of the Missed Seizure Rate and Management at Northamptonshire Healthcare NHS Foundation Trust (NHFT) Electroconvulsive Therapy Clinic

**DOI:** 10.1192/bjo.2024.606

**Published:** 2024-08-01

**Authors:** Sanaa Moledina, Jaiker Jani

**Affiliations:** Northamptonshire Healthcare NHS Foundation Trust, Northampton, United Kingdom

## Abstract

**Aims:**

The audit aimed to study missed seizure frequency, management, and restimulation rate at NHFT's ECT clinic.

**Methods:**

We conducted a retrospective analysis of ECT treatments administered between October 1, 2021, and March 21, 2023, collecting data on stimulation frequency and doses, duration of motor seizures and EEG activity, and patients' demographics. The study compared current practice with the NHFT ECT protocol, which defines missed seizures as treatments failing to induce convulsions and EEG activity. Management entails restimulation at least once or twice according to the stimulus dosing protocol during the seizure-threshold (ST) determination phase or by increasing the dose by 10% (50 millicoulombs) during the treatment phase, alternatively recording reasons for not re-stimulating. The ratio of missed seizures to total stimulations was used to determine the missed seizure rate, and the ratio of total restimulations to missed seizures was used to calculate the restimulation rate.

**Results:**

The clinic provided 268 treatment sessions and 26 courses of bilateral ECT to 23 patients aged 17–84 years, primarily female (60%) and Caucasian (74%), with a 12.6% missed fit rate and a 67.5% restimulation rate. Thirty missed seizures occurred during the initial ST determination phase, with twenty-two restimulated. Four of these could not be restimulated due to the maximum limit of three stimulations per ECT session. Seven missed seizures occurred later in the treatment phase, with three restimulated. For restimulations during the seizure-threshold determination phase, only eight of the twenty-two restimulation doses matched the stimulus dosing chart, and over half of these patients were stimulated at a lower-than-recommended dose. Once a seizure was generated and the threshold was identified, suboptimal maintenance doses were chosen, with 47% of patients stimulated on the same dose and 37% on doses only marginally over the ST in consecutive sessions. During the treatment phase, two out of three restimulations were performed with a dose lower than the specified 10% increase. The reasons for deviating from the guidelines were not documented.

**Conclusion:**

National audits of ECT clinics in 1981 and 1992 showed 50% and 25% missed seizure rates, respectively. Bridgend ECT Clinic maintained a missed fit rate of ≤5% over a 6-year period, which is half that of NHFT. Missed seizures have been associated with treatment failure and post-ECT adverse effects; hence, to effectively manage them, we propose that all ECT administration personnel be familiar with the NHFT ECT protocol, including the stimulus-dosing protocol, and document any clinical grounds for deviations.

